# Household Location (Urban, Peri-Urban and Rural Settlements) as an Associated Risk Factor for Toxoplasmosis during Pregnancy in Southeastern Brazil

**DOI:** 10.3390/tropicalmed9080173

**Published:** 2024-08-01

**Authors:** Maria Linda Ferreira Lima, Ana Maria Anthônia Ferreira Lima Simão de Sousa, Lucimara Lopes Marques, Isabella Braghin Ferreira, Rogério Giuffrida, Louise Bach Kmetiuk, Alexander Welker Biondo, Vamilton Alvares Santarém

**Affiliations:** 1 Graduate College in Health Sciences, University of Western São Paulo (UNOESTE), Presidente Prudente 19067-175, São Paulo, Brazilvamilton@unoeste.br (V.A.S.); 2 Medical School, University of Western São Paulo (UNOESTE), Presidente Prudente 19067-175, São Paulo, Brazil; 3 Laboratory LaborServ Pasteur, Mirante do Paranapanema 19260-000, São Paulo, Brazil; 4 Graduate College in Animal Sciences, University of Western São Paulo (UNOESTE), Presidente Prudente 19067-175, São Paulo, Brazil; 5 Zoonoses Surveillance Unit, Municipal Secretary of Health, Curitiba 81265-320, Paraná, Brazil; 6 Department of Veterinary Medicine, Federal University of Paraná (UFPR), Curitiba 80060-000, Paraná, Brazil

**Keywords:** epidemiology, parasite, social vulnerability, *Toxoplasma gondii*, zoonosis

## Abstract

Background: Brazil has a high prevalence of toxoplasmosis. However, there is a gap in comparing seroprevalence for *Toxoplasma gondii* across different environments, particularly among pregnant residents of rural and urban areas. Methods: The prevalence of IgG and IgM for *T. gondii* was compared among pregnant residents of the urban, peri-urban, and rural settlement areas in a municipality in southeastern Brazil. Information regarding age and area of residence was compiled from January 2015 to December 2022. Logistic regression analysis was used to assess the age and area of residence as risk factors. Results: A total of 1614 examinations were recorded, revealing 54.0% seropositivity, which was highest in the rural settlement (61.1%), followed by the peri-urban area (55.9%), and lowest in the urban area (49.2%). Conclusions: The high prevalence of IgG and presence of IgM in pregnant residents of rural, peri-urban, and urban areas highlights the significance of the results obtained for strengthening maternal health programs aimed at preventing toxoplasmosis, regardless of their residence.

## 1. Introduction

According to the Centers for Disease Control and Prevention in the United States, toxoplasmosis is among the six parasitic zoonoses prioritized for public health actions [1]. The disease is caused by *Toxoplasma gondii*, a protozoan whose felids may serve as definitive hosts and that may be able to infect any nucleated cell of warm-blooded animals [2]. The transmission of toxoplasmosis to humans primarily occurs through food ingestion, particularly raw or undercooked meat or partially washed fruits and vegetables. In addition, transmission can occur congenitally or through water-containing sporulated oocysts [3]. This disease is considered a primary cause of morbidity among food-borne illnesses [4,5].

The impact of toxoplasmosis may vary according to the inoculum quantity, the virulence of the infecting strain, host factors, such as immune status, and genetic history of individuals [6]. Although mainly asymptomatic in immunocompetent patients, the infection may affect the ocular system and several other systems. Ocular toxoplasmosis has been associated with retinitis due to alterations in secretion and growth factors, which may induce proliferation of epithelial cells, leading to a loss of visual acuity or permanent blind ness [7]. Toxoplasmosis can cause myocarditis [8], and cases with neurological involvement primarily include meningoencephalitis [9,10,11]. In immunocompromised individuals, toxoplasmosis can cause severe respiratory diseases [12]. 

Congenital transmission (congenital toxoplasmosis) can compromise fetal development [13] or induce neonatal alterations, primarily involving the eyes and the central nervous system, causing delays in mental development, cerebral palsy [14], and hearing disorders [15], and is considered a significant cause of miscarriage [14,16,17]. In Brazil, a meta-analysis identified retinochoroiditis and cerebral calcification as the most frequent findings in congenital toxoplasmosis [18]. 

Bigna et al. [19] estimated the overall prevalence of toxoplasmosis in pregnant women at 32.9% (95% CI: 29.4–36.4), with a notable emphasis on the Americas, exhibiting a higher prevalence at 45.2% (95% CI: 33.4–53.4). Rostami et al. [20] demonstrated that the prevalence of latent infection in pregnant women was 33.8% (95% CI: 31.8–35.9%; 345,870 out of 1,148,677), with higher prevalence in South America (56.2%; 50.5–62.8%). In Brazil, the toxoplasma prevalence in pregnant women has been varied from 50 to 80% [21], with no reduction in IgG or IgM seroprevalence worldwide [19]. In contrast, a decline in seroprevalence for toxoplasmosis has been observed in pregnant women living in high-income countries, such as France and Slovenia, and childbearing age women in Serbia and the Unites States, according to a recent review study [22].

The incidence of toxoplasmosis is significantly higher in women from low-income and low-human development index countries [20]. In Brazil, studies indicate some risk factors for toxoplasmosis, primarily low socioeconomic and/or educational levels and inadequate knowledge about this zoonosis [23,24]. A seroepidemiological survey involving the residents of a rural settlement in southeastern Brazil showed 52.5% seropositivity for *T. gondii* infection, and the identified risk factors included female sex and low monthly income [25].

In Brazil, a public free-of-charge health program has been established by the Brazilian Unified Health System for screening toxoplasmosis during women’s pregnancies. Despite this, no studies have involved comparing the prevalence and risk factors of toxoplasmosis in pregnant women from rural settlements with those from urban and peri-urban areas. Therefore, this study aimed to compare the prevalence and risk factors for toxoplasmosis in pregnant residents of urban, peri-urban, or rural settlements in southeastern Brazil using a spatial-temporal analysis between 2015 and 2022.

## 2. Materials and Methods

### 2.1. Study Design

This retrospective study involved collecting serological results for toxoplasmosis between January 2015 and December 2022. 

### 2.2. Study Area Characteristics

The Pontal do Paranapanema region, western São Paulo state, presented the largest number of rural settlements in Brazil at the time, with the municipality of Mirante do Paranapanema accounting for 31/168 (18.45%) of the total nationwide Brazilian settlements [26]. Mirante do Paranapanema has an estimated population of 18,415 in 2021 [27], with approximately 30% in rural areas, including the settlements. The municipality has a high rate of social vulnerability, accounting for 37.5% of the rural population. The average household income was approximately USD 255.00, with 31.2% of households below half of the minimum wage per capita [28].

Two peri-urban villages were part of the Mirante do Paranapanema municipality, including Cuiabá Paulista and Costa Machado, with approximately 1670 and 1630 inhabitants, respectively.

### 2.3. Data Collection

This study was conducted by surveying the data from an electronic database (Laborserv Pasteur Laboratory, Mirante do Paranapanema, São Paulo, Brazil), which included the anti-*T. gondii* antibody tests in pregnant residents of municipalities (urban, peri-urban, and rural settlements) between January 2015 and December 2022. This laboratory is responsible for investigating anti-*T. gondii* antibodies in a municipal population. Antibody testing for toxoplasmosis is a women’s health program (prenatal care) established by the Brazilian Ministry of Health.

### 2.4. Inclusion and Exclusion Criteria 

This study included the anti-*T. gondii* antibody tests recorded in the database for all pregnant women in the Mirante do Paranapanema municipality and assisted by the Brazilian Unified Health System, regardless of age and excluding non-residents. Accordingly, toxoplasmosis screening by IgM and IgG tests have been part of the prenatal screening established by the Brazilian Health Ministry, systematically performed during the first trimester of all pregnant women. In addition, follow-ups of pregnant women susceptible to infection or with suspected acute toxoplasmosis have been conducted by serology tests throughout the pregnancy.

### 2.5. Anti-T. gondii Antibody Screening 

The antibody screening was conducted using serum samples collected by the team at Laborserv Pasteur Laboratory. Detection of anti-*T. gondii* antibodies was performed by chemiluminescence immunoassay using commercial test kits for IgG (ABBOT Architect ToxoIgG. Ref.6C19, Wiesbaden, Germany) and for IgM (ABBOT Architect ToxoIgG. Ref.6C20, Wiesbaden, Germany).

### 2.6. Statistical Analysis

Data were firstly categorized and double-checked to exclude redundant or missing values. The continuous variable of age (years) was categorized based on quartiles. Following, the data were inserted in contingency tables and initially submitted to univariate analysis by chi-square test for assessing possible association among seropositivity and the independent variables. Finally, logistic regression (multivariate analysis) was performed after the univariate analysis, to assess the associated protective/risk factors to seropositivity. For this purpose, variables with *p*-value less than 0.2 in the univariate analysis were considered fit to be included in the logistic regression. Uni and multivariate analyses were performed by using the R software version 4.3.1 [29].

Analysis of temporal seropositivity distribution was made based on the monthly records of seropositive women to *T. gondii*, which were standardized using the direct method to represent the seropositivity rate, considering 10,000 women of childbearing age for calculation of the infection rate. The standardized data were used in constructing an autoregressive integrated moving average (ARIMA) model with a seasonal component using the “auto.arima” algorithm available in the forecast package of the R programming language [30]. The nonseasonal (p, d, q) and seasonal (P, D, Q) terms of the final model were determined after testing combinations that minimized the Akaike Information Criterion. The accuracy of the model was defined after conducting cross-validation and calculating accuracy measures, including scale-dependent errors (MAE = Mean Absolute Error, RMSE = Root Mean Squared Error), percentage errors (MAPE = Mean Absolute Percentage Error), and scaled errors (MASE = Mean Absolute Scaled Error) [31]. 

A significance level of 5% was adopted as the criterion for statistical significance.

## 3. Results

A total of 2165 tests for anti-*T. gondii* antibodies (IgG) were recorded between 2015 and 2022, excluding 551 (25.5%) from pregnant women who underwent more than one test during pregnancy. Thus, 1614 tests were included in this study.

Furthermore, 871/1614 (54.0%, 95% CI: 51.3–56.4) individuals were seropositive for IgG, with 410/833 (49.2%; 95% CI: 45.8–52.6) living in the urban area, 175/313 (55.9%; 95% CI: 50.4–61.3) in the peri-urban area, and 286/468 (61.1%; 95% CI: 56.6–65.4) in the rural settlements of Mirante do Paranapanema municipality.

Regarding the IgM antibody presence, 23/1614 pregnant women (1.43%; 95% CI: 0.95–2.1) tested positive, with 12/833 (0.014%; 95% CI: 0.008–0.025) living in the urban area, 5/313 (0.016%; 95% CI: 0.007–0.037) in the peri-urban area, and 6/468 (0.013%; 95% CI: 0.006–0.028) in the rural settlements. Out of the IgM seropositive pregnant women, 17/23 (73.9%) also presented anti-IgG antibodies. All pregnant women were of legal age, except for one 17-year-old girl (19–39, mean = 30.6 years).

The univariate and multivariate analyses were conducted to assess the association between the variables of residence (urban, peri-urban, and rural settlements), the age of pregnant women, and seropositivity for anti-*T. gondii* antibodies (IgG) (Table 1). 

Logistic regression revealed that the seropositivity (IgG) in pregnant residents of rural and peri-urban areas was 1.72 (95% CI: 1.36–2.17; *p* < 0.0001) and 1.36 (95% CI: 1.04–1.78; *p* < 0.023) times higher, respectively, than that of the residents of the urban area of the municipality. There was no significant difference (*p* = 0.181) between pregnant residents of rural and peri-urban areas. Similarly, there were no significant differences (*p* = 0.834) in the prevalence between the two peri-urban villages.

Regarding the association between seropositivity and the age of pregnant women (stratified by percentiles), logistic regression revealed that the odds of seropositivity were directly proportional to the increase in the age range of the pregnant women, reaching 3.17 (95% CI: 2.37–4.25; *p* < 0.0001) for those aged 31–45 years. 

Some Supplementary Information is given herein. The annual numbers of tests for anti-*T. gondii* antibodies (IgG) are presented in Supplementary Table S1. The prevalence decreased from 71.0% in 2015 to 48.2% in 2018. In 2018, the prevalence was approximately 50.0%. In addition, the number of test requests recorded between 2015 and 2018 increased. Subsequently, an average of 230 tests were conducted between 2019 and 2022, with the maximum number in 2021.

The age of the pregnant women (overall population) varied from 13 to 45 years (mean, 26.2; median, 26) (Supplementary Table S2). Among the pregnant women with positive results, the average age was 26.4 (range, 14–45) years, with no significant differences observed between the means obtained for the residents in the three locations (*p* = 0.817). The number of pregnant women aged <18 years varied from 13 to 17 years, representing 8.2% (132 of 1614) of the participants (Supplementary Table S3). The overall prevalence of seropositivity in this group was 41.7% (55 of 132; 95% CI: 33.6–50.2).

Automatic selection of the ARIMA model for the univariate time series was used to determine a model with one autoregressive (p = 1, d = 0, q = 0) and two seasonal (P = 2, D = 0, Q = 0) terms considering the monthly frequency of records of seropositive pregnant residents in the municipality (Supplementary Figure S1). However, the calculated terms were insignificant, indicating that the adjusted model could not capture the temporal and seasonal patterns in the data (Supplementary Table S4).

## 4. Discussion

To our knowledge, this is the first Brazilian study where the seroprevalence of toxoplasmosis among pregnant residents in the rural, peri-urban, and urban areas of a municipality are compared. The results (2015–2022) of their anti-*T. gondii* antibody detection were compiled from an electronic database related to the Mirante do Paranapanema municipality, which has the highest number of rural settlements in Brazil.

A high prevalence (54.0%) of IgG antibodies was observed herein. The rate was highest in the rural settlements (61.1%), followed by the peri-urban (55.9%) and urban (49.2%) areas, surpassing the global seroprevalence of latent infection in pregnant women estimated through meta-analyses, which showed a small variation from 32.9% (95% CI: 29.4–36.4) to 33.8% (95% CI: 31.8–35.9) [20].

Comparing the results herein with the estimated prevalence in Brazil (61.2%; 95% CI: 58.2–64.1) in a meta-analysis [20] revealed similarity to that observed in pregnant women from rural settlements and closeness to that found in those in the peri-urban areas. In Brazil, a high prevalence in pregnant women has been recorded in all regions, including the midwest (63.0% [32] and 89.0% [33]), northeast (63.3% [23] and 67.9% [34]), northern (71.0% [35]), southeast (49.5% [36]), and south (62.5% [37]), with low socioeconomic status being a primary risk factor [20,32].

In our study, the seropositivity among residents of rural and peri-urban areas was 1.62 and 1.31 times higher, respectively, than that of those in urban areas, corroborating studies in Nigeria, Africa [38] and Warsaw, Poland [39], where the highest seroprevalence occurred in pregnant residents of rural areas. The results suggest that a higher prevalence occurs in populations with lower incomes. The socioeconomic, demographic, and health conditions of families in rural settlements constitute a context of social vulnerability, as observed in settlements in the same region of São Paulo [40]. The low family income of settlers is considered responsible for a limited variety in diet and a high prevalence of food and nutritional insecurity [41]. In a study involving the adult population (*n* = 194) of a rural settlement in the Mirante do Paranapanema municipality, São Paulo, female sex and low monthly income were identified as risk factors for toxoplasmosis [25]. 

Herein, it was observed that the prevalence was directly proportional to age, with 3.34 times higher odds of a pregnant woman aged 31–45 years being seropositive than those aged 13–20 years. This phenomenon has also been observed in Rio de Janeiro, Brazil [42]. In Salvador, Bahia, pregnant women aged 35–44 years had 2.26 times higher odds of being seropositive than those aged 15–24 [43]. Similarly, in northeastern Brazil, the odds ratio was 3.81 (95% CI: 1.94–7.48) higher for pregnant women aged >25 years [34]. In Romania, there was a trend toward a higher prevalence with increasing age, being higher in pregnant women aged 31–41 years [44]. This study argues that continuous exposure to risk factors for toxoplasmosis may cause a high prevalence in pregnant women of older age, which could be adopted to justify our results.

The prevalence of IgM antibodies herein (1.43%) was lower than the 2.8 and 5.3% observed in the northeast [45] and north [32] of Brazil, respectively; however, it is close to the 1.5% observed in another study in northeastern Brazil [35] and the estimates for the global population of pregnant women at 1.1% [46] and 1.9% [19] revealed through meta-analyses. In contrast to what was observed for IgG, the prevalence of IgM was close for pregnant residents of urban areas, peri-urban residents, and rural residents. These data show that despite living conditions being more favorable for exposure to *T. gondii* oocysts in soil and water in rural areas, our results should be carefully interpreted. The presence of IgG and IgM in pregnant residents of urban areas may indicate their exposure and may also be associated with consuming or handling raw meat or inadequate food hygiene, as demonstrated by other studies on pregnant women in Brazil [32,35,47]. Another possibility is the population of owned and stray felines, which may not have adequate care conditions in both situations. As the first class of antibodies, frequently detected after primary infection and gradually declining during exposure, IgM antibodies have been mostly associated with acute cases; meanwhile, IgG usually rises somewhat later and may persist for long periods or lifetime and is thus associated with chronical cases [48].

The results obtained herein indicate a considerable increase in the number of examination records of pregnant women (124 in 2015 and more than 220 from 2018 onwards). In contrast, there was a reduction of approximately 71.0% in prevalence in 2015 to approximately 50% in the last 5 years. Decrease of toxoplasmosis seroprevalence in pregnant women living in high-income countries has been associated with risk factors such as health and education, prenatal screening, along with water source, sanitation, and hygiene [22]. Herein, taking into consideration the social vulnerability of pregnant women, a decline in seroprevalence could be a result of the adherence of such a population to the prenatal diagnostic screening program, adopted by the Brazilian Unified Health System. In addition, over 72% of pregnant women in Niteroi, southeast Brazil [49] and 55.6% in Maranhão, northeast Brazil [47] have reported no knowledge about toxoplasmosis. In Rio Grande do Sul, southern Brazil, 55.7% of pregnant women reported having knowledge about the disease, and most of them (53.7%) received the first information during prenatal care [50]. The adherence to prenatal care as a reduction cause may be more likely, as only one result was considered for pregnant women who underwent multiple tests during pregnancy, avoiding result duplication and prevalence overestimation. Nevertheless, the increased number of tests over time and the new inclusion of potentially less infected parts of the population in the screening program should be considered as plausible reasons for the decreased prevalence observed herein.

As limitations, the study herein was based on database information of pregnant women assisted by the Brazilian Unified Health System, which missed the personal information of residents living in rural and urban areas. Thus, along with the general database, further studies should also assess the personal information, probably by individual calls and including educational background and living environment. In addition, future surveys should also consider gestational age and avidity index tests to differentiate between chronic or primary infection, which all should be tested as associated risk factors for toxoplasmosis seropositivity.

## 5. Conclusions

The high prevalence of IgG and the presence of IgM in pregnant residents of rural, peri-urban, and urban areas highlights the significance of the results obtained for strengthening maternal health programs aimed at preventing toxoplasmosis, regardless of their residence.

## Figures and Tables

**Table 1:** Risk factors associated with the presence of anti-*Toxoplasma gondii* antibodies (IgG) in pregnant residents (*n* = 1614) of the urban, rural, and peri-urban areas of the Mirante do Paranapanema municipality, São Paulo, Brazil, between 2015 and 2022, through univariate and multivariate analyses.

	*T. gondii* Antibodies		Univariate Analysis		Multivariate Analysis	
	Positive (%)	Negative (%)	OR (95% CI)	*p*-Value	OR (95% CI)	*p*-Value
Variables	871 (54.0)	743 (46.0)				
Area of residence				<0.001		
Urban	410/871 (47.1)	423/743 (56.9)	1.0 (Reference)		1.0 (Reference)	
Rural settlement	286/871 (32.8)	182/743 (24.5)	1.62 (1.29–2.04)		1.72 (1.36–2.17)	<0.0001
Peri-urban	175/871 (20.1)	138/743 (18.6)	1.31 (1.01–1.70)		1.36 (1.04–1.78)	0.023
Age (years old) ^a^				<0.001		
13–20	143/871 (16.4)	213/743 (28.7)	1.0 (Reference)		1.0 (Reference)	
21–25	201/871 (23.1)	205/743 (27.6)	1.46 (1.09–1.50)		1.47 (1.1–1.96)	0.0094
26–30	230/871 (26.4)	180/743 (24.2)	1.90 (1.43–2.54)		1.96 (1.47–2.62)	<0.0001
31–45	297/871 (34.1)	145/743 (19.5)	3.04 (2.28–4.08)		3.17 (2.37–4.25)	<0.0001

^a^: age is distributed in percentile.

## Data Availability

The data presented in this study are available on request from the corresponding author. The data are not publicly available due to the request of third part where data were obtained.

## Supplementary Materials

The following supporting information can be downloaded at: https://www.mdpi.com/article/10.3390/tropicalmed9080173/s1, Figure S1. Location of the Mirante do Paranapanema region, Western São Paulo, Brazil. Table S1. Total number and results of *Toxoplasma gondii* antibody (IgG) detection in pregnant women (*n* = 1614) between 2015 and 2022 in the urban areas, peri-urban areas, and rural settlements of Mirante do Paranapanema, São Paulo, Brazil. Table S2. Mean and variation in the age of pregnant women (*n* = 1614) who underwent tests for detecting *Toxoplasma gondii* antibodies (IgG) between 2015 and 2022, living in the urban, peri-urban, and rural settlements of Mirante do Paranapanema, São Paulo, Brazil. Table S3. Distribution of the number and results of tests for the detection of *Toxoplasma gondii* antibodies (IgG) between 2015 and 2022 in pregnant women aged <18 years in the urban, peri-urban, and rural settlements of Mirante do Paranapanema, São Paulo, Brazil. Table S4. Temporal model for assessing the monthly incidence of positive tests for toxoplasmosis in pregnant residents of Mirante do Paranapanema, São Paulo state, between 2015 and 2022.
