# Supplementary material for: Household Location (Urban, Peri-Urban and Rural Settlements) as an Associated Risk Factor for Toxoplasmosis during Pregnancy in Southeastern Brazil
Source: Trop Med Infect Dis. 2024 Aug 1;9(8):173. doi: 10.3390/tropicalmed9080173 (PMC11358893; doi:10.3390/tropicalmed9080173)
Supplement: Supplementary file 1 [file tropicalmed-09-00173-s001.zip › tropicalmed-3051867-supplementary.pdf]

Tropical Medicine and Infectious Disease

MDPI

Supplementary Table S1. Total number and results of *Toxoplasma gondii* antibody (IgG) detection in pregnant women (n=1,614) between 2015 and 2022 in the urban areas, peri-urban areas, and rural settlements of Mirante do Paranapanema, São Paulo, Brazil

Result	Year								
	**2015**	**2016**	**2017**	**2018**	**2019**	**2020**	**2021**	**2022**	**Overall**
**Positive**	88	102	105	107	112	111	132	114	871
**Negative**	36	44	97	115	110	112	112	117	743
**Total**	124	146	202	222	222	223	244	231	1614
**Prevalence (%)**	71.0	69.9	52.0	48.2	50.5	49.8	54.1	49.4	54.0

Supplementary Table S2. Mean and variation in the age of pregnant women (n=1,614) who underwent tests for detecting *Toxoplasma gondii* antibodies (IgG) between 2015 and 2022, living in the urban, peri-urban, and rural settlements of Mirante do Paranapanema, São Paulo, Brazil

**Year**									
**Local**	**2015**	**2016**	**2017**	**2018**	**2019**	**2020**	**2021**	**2022**	**Overall**
**Urban**									
Mean	25.6	24.9	25.9	26.2	25.9	26.3	28.1	28.2	26.4
Variation	15-39	15-37	16-38	14-40	14-43	14-44	13-44	14-45	13-45
**Peri-urban**									
Mean	24.1	26.1	24.6	27.5	25.8	26.0	26.8	27.3	26.0
Variation	15-41	15-41	14-36	16-40	16-42	15-39	15-41	16-42	15-42
**Rural settlement**									
Mean	26.1	25.2	25.4	24.4	27.3	27.7	26.7	26.3	26.1
Variation	15-42	16-38	15-38	15-38	15-41	15-39	15-44	16-40	15-44
**Oveall**									
Mean	25.3	25.3	25.5	25.9	26.3	26.6	27.5	27.5	26.2
Variation	15-42	15-41	14-38	14-40	14-43	14-44	13-44	14-45	13-45
**Seropositives**									
Mean	25.6	25.4	26.7	27.2	28.2	27.9	29.3	28.8	26.4
Variation	15-42	15-41	15-38	14-40	14-43	15-44	17-44	16-45	14-45

Supplementary Table S3. Distribution of the number and results of tests for the detection of *Toxoplasma gondii* antibodies (IgG) between 2015 and 2022 in pregnant women aged <18 years in the urban, peri-urban, and rural settlements of Mirante do Paranapanema, São Paulo, Brazil

**Result**	**Year**								
	**2015**	**2016**	**2017**	**2018**	**2019**	**2020**	**2021**	**2022**	**Overall**
**Positive**	10	11	10	9	5	5	1	4	55
**Negative**	0	3	11	11	13	9	14	16	77
**Total**	10	14	21	20	18	14	15	20	132
**Prevalence (%)**	100	78.5	47.6	45.0	27.8	35.7	6.7	20.0	41.6

Supplementary Table S4. Temporal model for assessing the monthly incidence of positive tests for toxoplasmosis in pregnant residents of Mirante do Paranapanema, São Paulo state, between 2015 and 2022.

Component	Coef.	s.e.	Z	p
ar1	0.172	0.109	1.574	0.116
sar1	-0.213	0.109	-1.95	0.052
sar2	0.181	0.111	1.626	0.104

Term set for the ARIMA model: (1,0,0)(2,0,0)[12].
ar = non-seasonal autoregressive component; sar = seasonal autoregressive component; Coef = coefficients of the ARIMA model; s.e. = standard error; Z = Z-statistic; p = significance value of the z-statistic for the hypothesis that the coefficient differs from zero; AIC=544.21; BIC (Bayesian Information Criterion) = 557.04; MAE= 3.12; RMSE = 3.87; MAPE = 30.17; MASE = 0.56

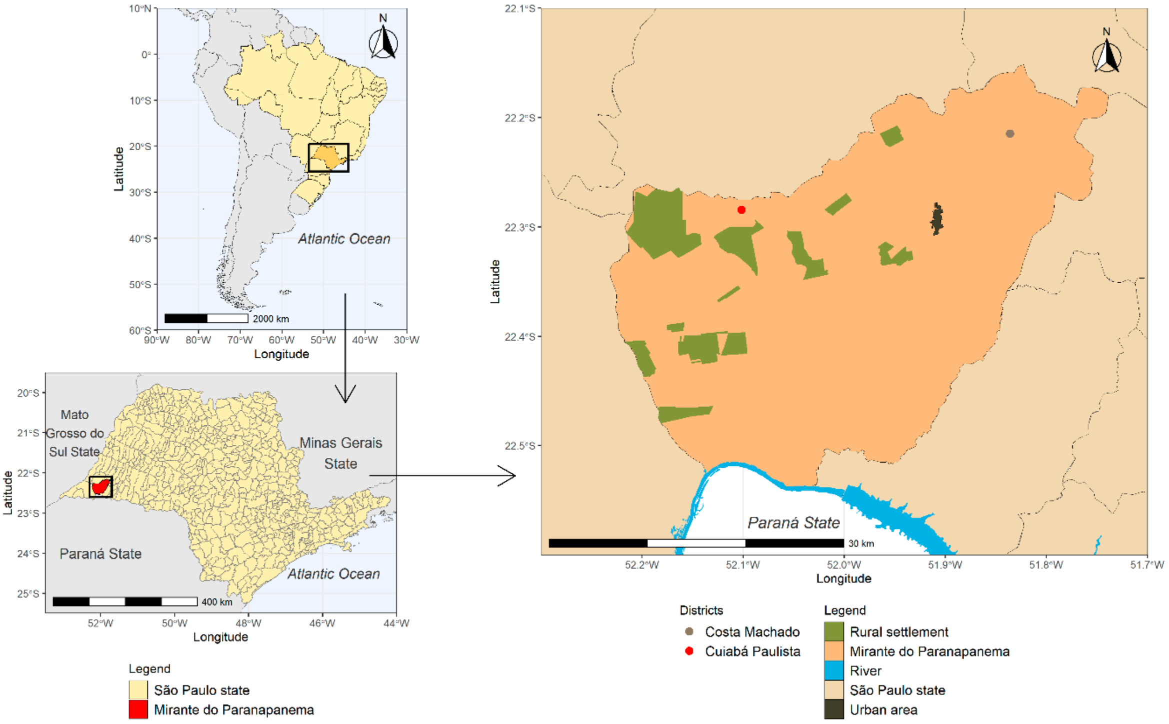

Supplementary Figure S1. Location of the Mirante do Paranapanema region, Western São Paulo, Brazil.
